# HTS-PEG: A Method for High Throughput Sequencing of the Paired-Ends of Genomic Libraries

**DOI:** 10.1371/journal.pone.0052257

**Published:** 2012-12-20

**Authors:** Sisi Zhou, Yonggui Fu, Jie Li, Lingyu He, Xingsheng Cai, Qingyu Yan, Xingqiang Rao, Shengfeng Huang, Guang Li, Yiquan Wang, Anlong Xu

**Affiliations:** 1 State Key Laboratory of Biocontrol, Guangdong Province Key Laboratory of Pharmaceutical Functional Genes, Department of Biochemistry, College of Life Sciences, Sun Yat-Sen University, Higher Education Mega Center, Guangzhou, The People’s Republic of China; 2 Key Laboratory of the Ministry of Education for Coastal and Wetland Ecosystems, School of Life Sciences, Xiamen University, Xiamen, China; Auburn University, United States of America

## Abstract

Second generation sequencing has been widely used to sequence whole genomes. Though various paired-end sequencing methods have been developed to construct the long scaffold from contigs derived from shotgun sequencing, the classical paired-end sequencing of the Bacteria Artificial Chromosome (BAC) or fosmid libraries by the Sanger method still plays an important role in genome assembly. However, sequencing libraries with the Sanger method is expensive and time-consuming. Here we report a new strategy to sequence the paired-ends of genomic libraries with parallel pyrosequencing, using a Chinese amphioxus (*Branchiostoma belcheri*) BAC library as an example. In total, approximately 12,670 non-redundant paired-end sequences were generated. Mapping them to the primary scaffolds of Chinese amphioxus, we obtained 413 ultra-scaffolds from 1,182 primary scaffolds, and the N50 scaffold length was increased approximately 55 kb, which is about a 10% improvement. We provide a universal and cost-effective method for sequencing the ultra-long paired-ends of genomic libraries. This method can be very easily implemented in other second generation sequencing platforms.

## Introduction

Next generation, massively parallel sequencing, notable for its high throughput and cost-efficiency, has accelerated the pace of genome sequencing of new species. Millions, even billions, of reads are generated in a single instrument run, but the sequence read length it obtained is shorter than that of the traditional Sanger method [1], [2]. Paired-end sequencing first emerged as a key technical strategy to order and orient contigs into scaffolds [3]. Various spans of paired end reads are used to resolve assembly of the shotgun reads into a fine map of the genome [4], [5], [6], [7]. Procedures for generating paired-end/mate pair libraries for second generation sequencing platforms are commercially available, such as the Roche GS FLX Titanium, Illumina GA XII and ABI SoLid. In each of these methods, the main workflow includes DNA fragmentation, circularization, random shearing/enzyme digestion, adaptor ligation and amplification [8], [9], [10]. The larger the span of a paired-end library, the lower the level of uniformity of the fragments and the lower the efficiency of circularization. The spans of these libraries are limited and usually range from 800 bp to 20 kb, sometimes up to 40 kb, which depend on the size of the first fragmentation.

A genomic library is constructed by a cloning vector that carries exogenous DNA fragments. Different types of genomic libraries are distinguished by their vectors, each with different characteristics and capacities. Hong [11] reported the first paired-end sequencing method using a genomic library with the traditional Sanger capillary sequencing method, which provided an excess of mapping coverage and contributed significantly to scaffold assembly [12], [13], [14]. This method is still used to sequence the paired-ends of genomic libraries, such as Bacteria Artificial Chromosome (BAC)/P1-derived Artificial Chromosome (PAC), whose span could be over 300 kb. Both the construction of genomic library and sequencing the paired-ends with the Sanger method are expensive and time-consuming. Recently, two methods for creating terminal tags of genomic libraries have been developed for high-throughput sequencing. However, one of these methods is limited to its read length and needs additional data analysis to identify the read pairs [15], and the other is limited to specific vectors [16], [17].

**Figure 1:**
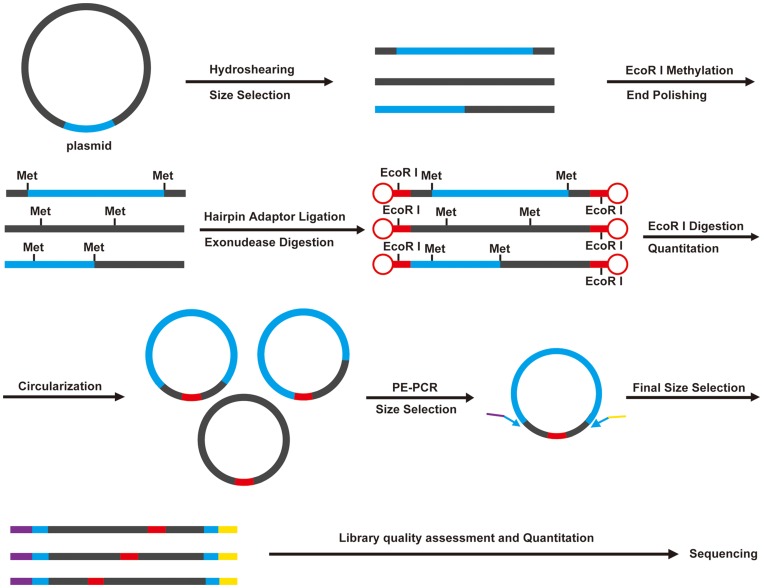
Flow chart illustrating HTS-PEG. The plasmids of the genomic library were sheared to yield fragments of 100–1500 bp larger than the vector (Blue). Then, the EcoR I sites were methylated and hairpin adaptors (Red) which contain non-methylated EcoR I sites were ligated to the fragment ends. After EcoR I digestion and circularization, the paired ends can be amplified by primers that are complementary to the ends of the vector. The PCR products with the desired size can be sequenced using the high-throughput sequencing method.

Here we developed a novel and universal high throughput method to sequence the paired-ends of a genomic library as an ultra-long span paired-end library preparation method (Figure 1). Our method does not require the isolation of clones while constructing the BAC library; directly culturing the transformation products is possible. We applied this method to construct a paired-end library using a Chinese amphioxus (*Branchiostoma belcheri*) BAC library [18] and sequenced it with 454 sequencing technology. The results significantly improved the genome assembly of Chinese amphioxus. This method could be easily implemented in other second generation sequencing platforms.

## Results

### Overview of BAC Paired-end Library Preparation Procedure

In this procedure, BAC clones are cultured separately and pooled together to extract the plasmids. Next, the plasmids are fragmented by hydrodynamic shearing, and the size distribution of the fragments are kept within ±1 kb of the vector backbone of the genomic DNA library. EcoR I site methylation is introduced to protect the fragments from EcoR I cleavage. After fragment end polishing, hairpin adaptors containing a non-methylated EcoR I site are ligated to the ends of the fragments, and subsequently all the fragments that are not ligated by hairpins are digested by exonucleases. The hairpin structures are removed by EcoR I digestion, and the cohesive ends, which can be used to self-circularize the linear fragments, are released. In the paired-end-PCR (PE-PCR) step, chimeric primers are designed with the 5′ portion containing the sequencing primer A/B, and the 3′ portion containing a template-specific primer that is complementary to each end of the linearized vector. Then, the self-circularized DNA containing the complete vector can be amplified. After library quality assessment and quantitation, the paired-ends of the genomic library can be sequenced with 454 sequencing technology (Figure 1).

### Sequencing the Paired Ends of a BAC Library of Chinese Amphioxus

**Figure 2:**
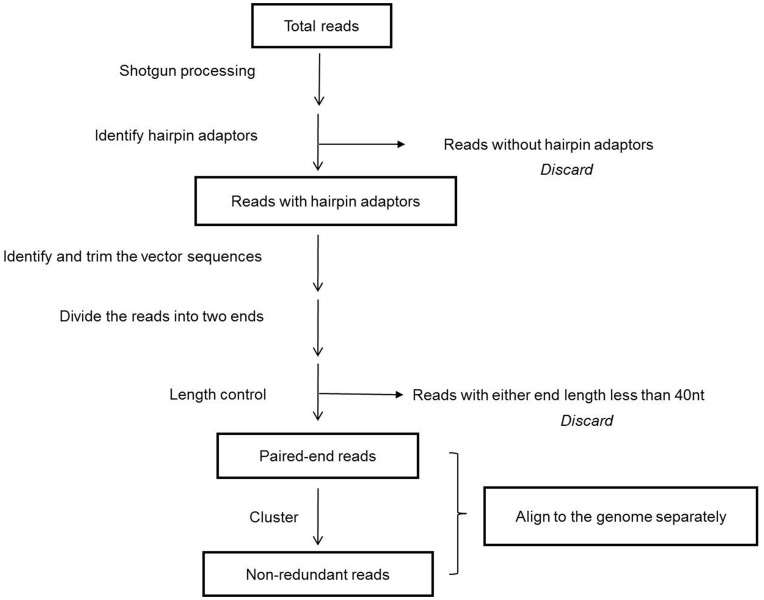
The workflow of data processing. The raw data were first filtered with the hairpin adaptor; reads without hairpin adaptor sequences were discarded. Then the vector sequences of the remaining reads were trimmed. The reads were then divided into left and right ends, and those reads with either end length less than 40 nt were discarded. The filtered paired-end reads were clustered and mapped to the genome.

**Figure 3:**
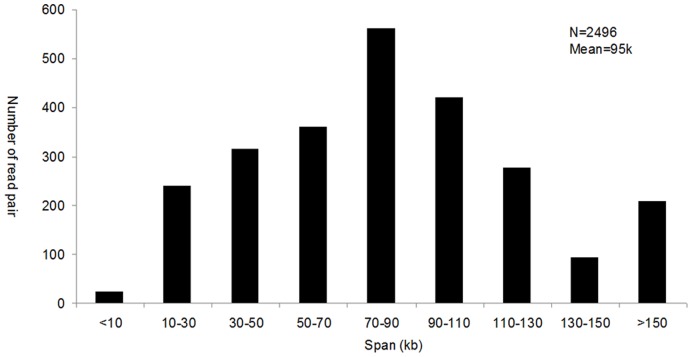
Span distribution of the non-redundant read pairs mapped to the Chinese amphioxus genome.

We used this method to construct a paired-end library of a Chinese amphioxus (*Branchiostoma belcheri*) BAC library [18], which was sequenced with 454 sequencing technology. The raw data were processed as follows (Figure 2): i) identify the hairpin adaptor sequences and filter the reads that lacked hairpin adaptor sequences; ii) find and trim the vector sequences; iii) divide the reads into two ends and discard pairs for which either end is less than 40 nt; iv) cluster the resulting paired-end reads to obtain non-redundant reads; and v) align the read pairs to the Chinese amphioxus genome (Huang et al., unpublished data). In total, approximately 1.2 million raw reads were obtained, and among those, 634,086 reads were identified as true paired-ends with the hairpin adaptor sequences. Among the 443,353 pairs with both ends longer than 40 nt, 91.5% were full length and could be identified by both 5′ and 3′ vector sequences. To obtain non-redundant data, the read pairs were clustered, and 12,670 non-redundant pairs were generated (Table 1). Of these non-redundant read pairs, 2,496 paired BAC ends could be mapped to the same scaffold, and the average span was approximately 95 kb (Figure 3), which is consistent with the previous report [18]. The incidence of chimeric pairs with the wrong orientation is estimated at approximately 6.85% (Table 1).

**Table 1:** Summary of the HTS-PEG data.

	Number
Total raw data reads	1,234,870
Reads with hairpin adaptor sequences	634,086
Read pairs with both ends larger than 40nt	443,353
Full length read pairs with both ends larger than 40nt	404,912
Non-redundant read pair clusters	12,670
Non-redundant full length read pair clusters	9,409
Read pairs mapped to multiple genomic locations	111,570
Read pairs mapped to a unique genomic location	178,399
Read pairs mapped to the same scaffold	101,914
Read pairs mapped to different scaffolds	188,055
Non-redundant read pairs mapped to a unique genomic location	4,569
Non-redundant read pairs mapped to the same scaffold	2,496
Non-redundant read pairs mapped to different scaffolds	4,979
Chimeric read pairs with wrong orientation	6,986

### Sequencing Saturation Simulation

**Figure 4:**
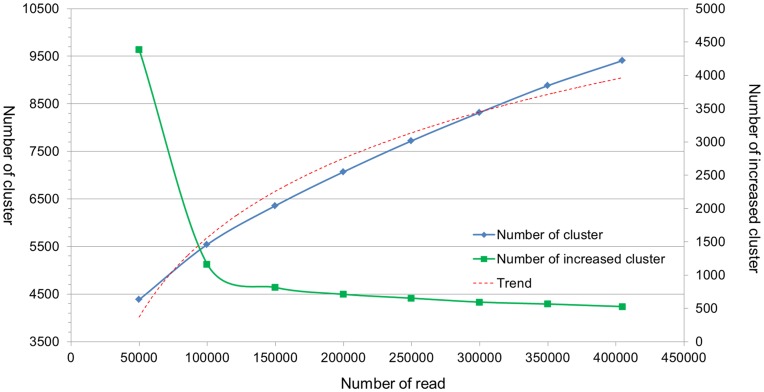
Increasing tendency of non-redundant read pairs. 50,000, 100,000, 150,000, 200,000, 250,000, 300,000 and 350,000 read pairs were randomly selected and clustered, and three replicates were made at each sampling size. Blue line represents the observed number of cluster, while red line represents its trend. And green line represents the number of increased cluster.

To monitor the saturation level of the sequencing, we randomly selected different numbers of read pairs that were full length, calculated their cluster number and increased cluster number, and obtained the increased tendency of non-redundant reads (Figure 4). The trend shows that after 300,000 reads, the increasing rate tends to be very low, which indicates that the present paired-end library was nearly fully sequenced, and another paired-end library should be reconstructed and sequenced to obtain additional read pairs.

### Mapping and Assembling the Genome of Chinese Amphioxus

In our effort to sequence Chinese amphioxus *de novo* (Huang et al., unpublished observations) with 454 and Illumina sequencing techniques, we used this new strategy to improve the assembly quality of the genome. We first mapped the read pairs to the primary assembled genome, which includes 11,522 scaffolds with a total length of 504 M bases. Among the 443,353 read pairs, approximately 65.4% could be mapped to the genome with both ends, and 29.5% of them had only one end mapped, possibly owing to the current incomplete genome or the diversity of Chinese amphioxus (Figure S1 and Table 1). Approximately 35.1% of reads with both ends mapped to the genome were mapped to the same scaffold. The read pairs that mapped to different scaffolds were used to assemble the genome. In total, these paired ends were mapped to 1,182 scaffolds, and then 413 super-scaffolds were assembled. The scaffold N50 length increased from 541,615 to 596,166 bp after this improvement (Table 2).

**Table 2:** Improved scaffold length of the Chinese amphioxus genome using the paired BAC ends.

	Raw assembly of Chinese amphioxus genome	Using paired BAC ends
Number of scaffolds	11,522	10,753
Span (bp)	504,005,601	504,082,501
N50 scaffold (bp)	541,615	596,166

## Discussion

Second generation sequencing technology makes *de novo* sequencing of non-model organism genomes practical and rapid, but a key obstacle to obtain a good genome is the assembly of longer scaffolds for better analyses of gene annotation, synteny and evolution. Previously, various methods were developed to construct paired-end (or mate pair) libraries, but their span is usually less than 20–40 kb. The classical BAC and fosmid libraries with the insertion fragments of more than 40 kb have typically been used to provide the ultra-long paired-end sequences with the Sanger sequencing method; however, this method is time-consuming and expensive. Recently, enzyme digestion and ShARC methods have been developed to generate two terminal tags of a fosmid/BAC library with the second-generation sequencing method [15], [17]. However, the method of enzyme digestion required a specific vector that contained Illumina or 454 sequencing primer binding sites flanking the insert cloning site. The ShARC method used blunt end ligation during the re-circularization step, without any adaptors that can distinguish the two termini of the genomic fragments. Thus, the paired-ends cannot be easily identified, which necessitates additional processing; furthermore, the read lengths were limited. Here we reported a universal and accurate method for sequencing the paired-ends of genomic libraries in a high-throughput manner.

### The Characteristics of HTS-PEG

In contrast to the previous paired-end or mate pair libraries, the span of the paired-end library generated by our method, based on a BAC or fosmid, is dependent upon the inserts of the raw genomic library instead of the size of the hydrodynamic sheared fragments. For a fosmid library, the average insertion length is approximately 20–45 kb, and for a BAC/PAC library, its capacity is approximately 100–300 kb. Regardless of the length of the inserted size of the library, it is only necessary to shear the plasmids to the size range that is 100–1000 bp larger than its vector, which is usually 6–15 kb. Then we can easily obtain paired-ends spanning even 300 kb. In the re-circularization step, we introduced a hairpin adaptor to separate the two termini of the genomic fragments, thus making it easy to discern the true or false paired-ends during data processing. In the PCR step, a pair of chimeric primers was used, of which the 5′ portion contains the GS FLX sequencing primer A/B and the 3′ portion contains the template-specific primer that is complementary to each end of the linearized vector. In this way, we can amplify the two terminals of the genomic library and ligate the sequencing primers simultaneously, which makes the procedure much easier to perform.

In our proof of principle experiment, we used a Chinese amphioxus (*Branchiostoma belcheri*) BAC library. This library was constructed with individually picked clones, but we pooled all clones together to extract the plasmid simultaneously when conducting the experiment. Therefore, the situation is quite similar to the non-isolated clones. Although the BAC library we used is a well-constructed library with individual clones, it was not screened for the positive clones, and contained approximately 12% negative BAC clones (without exogenous insert DNA). This is the same situation as with the non-isolated transformation products. The negative BAC clones are the key factors that would seriously impact paired-end library construction because they will be more easily amplified without inserts. To overcome this problem, we introduced double SPRI size selection during the paired-end PCR step. The amplicons of negative clones is a short fragment with the same fixed size, which is different from the target size that is amplified from the positive clones. AMPure beads were used to exclude the short fragments after the first 10 cycles of PCR to eliminate their interference with the amplification. Then, the following 10–15 cycles of PCR were performed to enrich the amplicons of the desired size range. In this way, we can eliminate the interference of negative clones that are problematic for the paired-end library construction. Therefore, even the non-isolated clones could be used directly to construct the paired-end library in our method. To select the size more precisely, after the second round of PCR, PAGE separation and extraction is recommended. If the genomic library used has already been screened for the presence of an insert and only the positive BAC plasmids are used, one round of amplification and size selection is sufficient.

### Chimeric Paired-ends

The percentage of chimeric paired-ends is a criterion to measure the quality of a library. Peng *et al* defined chimeric pairs as those that map to different chromosomes or in the wrong orientation [19]. Mapping to different chromosomes can here be considered as those pairs whose spans are distinctly different from the original size; the span of our paired-end library is depended on the original DNA insertion size in the BAC library. The digested DNA fragments in the 97–145 kb range size were extracted to construct the BAC library, and the average insertion size was estimated to be approximately 80 kb by measuring a random sampling of 76 clones from three 384-well plates [18]. We analysed the span distribution of read pairs that mapped to the same scaffold. The result showed that the average insertion size is approximately 95 kb, which is consistent with the previous report that demonstrated the accuracy of this method (Figure 3). We also estimated the percentage of chimeric pairs with the wrong orientation using the read pairs that mapped to the same scaffold. We found 6.85% in the wrong orientation, which is lower than the 8.5% reported from the 5 kb mate-pair library generated by the Illumina jumping method [19].

### Assembly Efficiency

The DNA sample we used to construct the BAC library was not from the DNA sample used for Chinese amphioxus sequencing because Chinese amphioxus is a small animal, and only limited DNA can be obtained from one individual. The diversity of amphioxus genome is approximately 0.0562 [20], which is very high compare to the diversity of human genome (less than 0.001) [21], [22]. Because of the high diversity of the amphioxus genome and the current incomplete genome, only 65.4% of read pairs mapped to the genome with both ends, of which 64.9% read pairs mapped to different scaffolds and were used to assemble the genome (Table 1 & Figure S1). The assumed genome size of Chinese amphioxus is approximately 575 Mb per haplotype, so the physical coverage we obtained is approximately 1.9×, which is not sufficient for efficient scaffolding. But, even in this circumstance, the N50 scaffold still had approximately 10% improvement, which showed the efficiency of this method compared to approximately 30% N50 scaffold improvement that resulted from 3 runs of 20 kb paired-end sequencing by 454 (Huang et al., unpublished observations).

### Trace Back to Single Clone

In this experiment, we focused on obtaining the paired BAC information using HTS-PEG to assemble the genome, thus all the clones were pooled together to construct the library without any marking. This method is quite cost-efficient compared to Sanger sequencing, but the obtained paired-end sequences cannot be traced back to the specific BAC clone. However, sometimes researchers need to get the information of a single specific clone to resolve this obstacle, we suggest here first pooling the clones into different groups, and then constructing paired end libraries from each group. In the PE-PCR step, complex primers with added MID sequences can be used to barcode each group. Thus, an individual clone can be traced back to one group, and can be further confirmed using Sanger sequencing. Another advantage of using this strategy is the final sequencing library would consist of multiple libraries. This reduces the redundancy of the sequencing library, and therefore, generates more non-redundant read pairs.

### Efficiency Improvement of HTS-PEG

All the clones were cultured separately and pooled to extract the plasmids. The mixed plasmids were used for library construction. There are several reasons that explain why only 12,670 read pairs were obtained. First, a previous report estimated that about 12% of clones, or approximately 5,365, do not have insertions in this library [18]. Second, the BAC library has redundancy. Third, the low amount of DNA used for circularization increased the library redundancy. And finally, there is amplification bias during the PE-PCR step. The last two points are the most important. For DNA circularization, using larger amounts of DNA would generate a more diverse library, but it would also elevate levels of chimeric reads owing to the liner ligation of two or more DNA molecules. Thus, we recommend performing several DNA circularization reactions in parallel using all DNA obtained from the digestion step. During the PE-PCR step, replicate reactions are also recommended to reduce the amplification bias.

To determine whether we can obtain more non-redundant read pairs by directly sequencing another run of 454 with this paired-end library, we performed a simulation to monitor the saturation level of the sequencing. By random selection and clustering of the reads, we can plot the increasing tendency of non-redundant pairs. If the curve is still in the phase of rapid growth, we can simply add another run of sequencing; however, if the increasing curve has reached plateau, a new paired-end library should be reconstructed and sequenced to obtain more non-redundant read pairs. From the simulation result, we could see that after 300,000 reads the increasing rate became very low, indicating that most of the information contained in this library was already obtained under current sequencing depth (Figure 4). If we continue sequencing this library, surely we could obtain some more non-redundant reads, but the efficiency would be quite low. Sequencing a new library would be a better choice than just continuing sequencing the current one. In this way, a high level of redundant sequencing could be avoided. Because redundancy is a common problem of paired-end libraries [23], [24], it is suggested in the 454 paired-end library preparation manual to construct 2 to 6 independent 8 kb paired-end libraries starting with no less than 15 µg of input DNA. Our data also indicated that using the mixture of multi-libraries is a better way to obtain more non-redundant read pairs.

The read length is an important factor in mapping and assembling the genome. We used the read pairs with the length of each end larger than 40 nt to guarantee the assembly quality. The average read length of 454 sequencing was approximately 350 nt when we did the experiment, which made the average read length of each end approximately 100–120 nt (Figure S2). Because the fragmentation is random, parts of reads were discarded as a result of one end being shorter than 40 nt. The newest version of 454 sequencing with an average read length of 600–800 nt is currently available. Thus, the length of each end can be elevated to 250–350nt, and the percentage of read pairs having both ends larger than 40 nt would be greatly increased. Therefore, we highly recommended using the upgraded 454 platform to sequence the library constructed by following this method.

Currently, the cost to generate a BAC two-terminal sequence by the Sanger method is approximately $2 in a large sequencing centre, and a full 454 run is approximately $10,000. Based on our experiment, the cost savings is over 2.5 fold despite the cost of the plasmid extraction. However, we can improve the efficiency of HTS-PEG to yield more useful information, as we discussed previously. First, replicate reactions should be performed in parallel during the circularization and PE-PCR steps to reduce the library redundancy and avoid amplification bias. Second, the sequencing library should be generated by mixing multiple libraries to maximize the library diversity. Finally, the new version 454 sequencing technology, which generates an average read length of approximately 600–800 nt, should be utilized. Because many reads were discarded due to the limited sequencing length, the upgraded 454 technique would certainly benefit our method. Thus, the cost of sequencing the paired-end of genomic library following HTS-PEG can be at least 10-fold lower compared to the traditional Sanger methods.

### Conclusions

In summary, this method provides a novel and universal procedure to construct ultra-long span paired-end libraries based on the classical genomic library. It is quite cost-efficient and very easy to implement for high-throughput sequencing the two termini of genomic libraries based on Illumina and ABI SoLid.

## Materials and Methods

### The Chinese Amphioxus (*Branchiostoma belcheri*) BAC Library

The BAC library was constructed using a single individual of Chinese amphioxus (*Branchiostoma belcheri*) [18]. This library consists of 44,706 clones, whose average insert fragment size is ∼80 kb, and the vector is the CopyControl™ pCC1BAC™ Vector (Epicentre, Madison, USA), which has a length of 8,128 bp.

### Plasmid Extraction and Hydroshearing

Clones of the BAC library were separately cultured in 96-well plates in LB medium in a 37°C shaker overnight and pooled together to extract the plasmids using a plasmid midi kit (Qiagen, Hilden, Germany). Approximately 40 µg of plasmid was hydrodynamically sheared using a standard assembly with setting of 15 for 15 cycles (HydroShear, Genomic Solutions). Because the vector is 8,128 bp, we cut the bands between 8 to 10 kb and purified them with a Qiaquick gel extraction kit (Qiagen, Hilden, Germany).

### EcoR I Site Methylation and Hairpin Adaptor Ligation

The EcoR I sites were methylated with 200U EcoR I methylase (NEB, Ipswich, MA) in 100 µl of 1× EcoR I methylase buffer and 0.16 mM S-adenosylmethionine (NEB, Ipswich, MA) at 37°C for 1 h. Methylated fragments were purified on a MinElute column (Qiagen, Hilden, Germany). Fragment ends were polished with 15U T4 DNA polymerase (Roche) and 50U polynucleotide kinase (Roche) in 50 µl of 1× PNK buffer, 0.1 mg/ml BSA (Roche), 1 mM ATP (Roche), and 0.4 mM PCR Nucleotide Mix (Roche) at 12°C for 15 min, and 25°C for 15 min. The fragments were purified using a MinElute column.

Polished DNA and hairpin adaptors (Roche) were ligated with 25U T4 DNA Ligase (Roche) in 100 µl of 1× T4 DNA ligation buffer at 25°C for 15 min. They were then digested with 10U Lambda exonuclease (NEB, Ipswich, MA), 20U T7 Exonuclease (NEB, Ipswich, MA), and 40U Exonuclease I (NEB, Ipswich, MA) at 37°C for 30 min. The hairpin adaptor-ligated DNA was purified on a Qiaquick column (Qiagen, Hilden, Germany). After the first spin, 700 µl of 8 M Guanidine HCl was added to the column to completely denature the exonucleases. Next, two PE washes were performed, and finally, the DNA was eluted in 100 µl of 10 mM Tris-Cl, pH 8.5. By adding 50 µl of AMPure beads (Beckman Coulter, Brea, CA) to the 100 µl of purified DNA solution, the hairpin adaptor ligated DNA was further selected and the small fragments were removed.

### EcoR I Digestion and Circularization

Removal of the hairpin structure was conducted in a 100 µl reaction with 1× SuRE/Cut buffer H, 200U restriction endonuclease EcoR I (high concentration) (Roche), and the hairpin adaptor-ligated DNA at 37°C for 16 hours. The reaction was purified with a QIAquick column. After the first spin, 700 µl of 8 M Guanidine HCl was added to the column to denature the enzyme. Next, two PE washes were performed, and finally, the DNA was eluted in 50 µl of 10 mM Tris-Cl, pH 8.5. The sample was quantitated using the Quant-iT PicoGreen dsDNA Assay kit (Invitrogen, Carlsbad, CA). Note that at least 30 ng is required to proceed with this method of quantitation.

DNA circularization was performed in three 200 µl reactions with 1× NEBuffer 4 (NEB, Ipswich, MA), 1 mM ATP, 30 ng EcoR I digested DNA and 25U T4 DNA Ligase at 25°C for 1 hour. 10U Lambda exonuclease, 20U T7 Exonuclease, and 40U Exonuclease I were then added to the reactions to digest the linear DNA at 37°C for 30 min. After purification on a Qiaquick column with 700 µl of 8 M Guanidine HCl to denature the exonucleases, the DNA circles were amplified.

### Paired-end Library Amplification and Size Selection

For the first round PCR, the reaction was in four 50 µl replicate tubes with 0.5× GC-RICH PCR reaction buffer, 3 mM MgCl_2_, 0.4 mM dNTPs, 2 µM each of primer A-Forward (5′-CGTATCGCCTCCCTCGCGCCATCAGTAATACGACTCACTAT- AGGG-3′) and primer B-Reverse (5′-CTATGCGCCTTGCCAGCCCGCTCAGTAC- GCCAAGCTATTTAGGTGAGA-3′), with 5U GC-RICH Enzyme mix (Roche). The PCR program was: 3 min at 94°C; 20 cycles of 30 sec at 94°C, 30 sec at 52°C, and 50 sec at 72°C; and 10 min at 72°C. Then, 50 µl of 10 mM Tris-Cl, pH 8.5 was added to each 50 µl reaction. The 100 µl solutions were transferred to 1.5 ml tubes containing 75 µl AMPure beads and incubated at room temperature for 5 min. Then the beads were pelleted, and the supernatant were transferred to new 1.5 ml tubes containing 85 µl AMPure beads and incubated at room temperature for 5 min. The beads were pelleted again, and the supernatant was discarded. Beads that bounded the desire size range amplicons were washed twice with 70% ethanol, and finally the amplicons were eluted with 10 µl of 10 mM Tris-Cl, pH 8.5.

All the DNA we obtained from the first round PCR and size selection were used as templates for the second round PCR. The reaction was also conducted in four 50 µl replicate tubes with 1× advantage 2 buffer, 0.2 mM D-(+)-trehalose dehydrate (Sigma), 0.4 mM dNTPs, and 2 µM each of primer A-Forward and primer B-Reverse, with 1U Advantage 2 polymerase (BD). The PCR program was 3 min at 94°C; 10–14 cycles of 30 sec at 94°C, 30 sec at 52°C, and 45 sec at 68°C; and 5 min at 68°C. The amplicons were finally size selected by electrophoresis on a 6% PAGE gel, and bands between 400–500 bp were excised, purified, and eluted in 10 µl of 10 mM Tris-Cl, pH 8.5.

### Library Quality Assessment, Quantitation and Sequencing

The size distribution of the BAC paired-end library was analyzed on a BioAnalyzer DNA 7500 chip (Agilent Technologies, Santa Clara, CA). The concentration of the library was quantitated using the Quant-iT PicoGreen dsDNA Assay kit. Finally, the library was sequenced with the GS FLX Titanium and amplicon emPCR kit.

### Trimming the Hairpin Adaptor, Vector Sequences and Filtering the Length

The GS References Mapper Application 2.3 with default settings was used to map the reads to the 44 nt hairpin adaptor sequences (5′- GTTGGAACCGAAAGGGTTTG AATTCAAACCCTTTCGGTTCCAAC -3′). After filter the reads without hairpin adaptor sequences, the remaining reads were then trimmed the primer and vector sequences with 5′ portion (5′- TAATACGACTCACTATAGGGCGAATTC -3′) and 3′ portion (5′- GAATTCGAGCTCGGTACCCGGGGATCCTCTAGAGTCGACC- TGCAGGCATGCAAGCTTGAGTATTCTATAGTCTCACCTAAATAGCTTGGCGT -3′). The trimmed sequences were then divided into left and right parts, and those having the length of both parts greater than 40 nt were kept as read pairs.

### Cluster Identification

Uclust (uclust3.0.617_i86 linux32) was used to obtain non-redundant paired-end reads. For all read pairs, the left and right sequences were sorted with decreasing length separately. Considering the presence of parts of reads which were not sequenced to their full length, we clustered the left end using Uclust with parameters: –gapext 20I –id 0.90–maxaccepts 0–maxrejects 0–allhits –nofastalign –iddef 2; and the right ends with parameters: –gapext 20I –id 0.90–maxaccepts 0–maxrejects 0–allhits –nofastalign, respectively. CD-HIT-454 was also used to validate the result (parameters: -c 0.9–b 20–M 0–g 1–D 2). Reads with both ends in the same cluster were considered as redundant read pairs and merged to one non-redundant pair.

### Sequencing Saturation Simulation

To determine the level of sequencing saturation and obtain more useful pairs, we plotted the growth curve of non-redundant read pairs by randomly selecting 50,000, 100,000, 150,000, 200,000, 250,000, 300,000 and 350,000 full length read pairs. CD-HIT-454 and Uclust were used to estimate the non-redundant read pairs. Each sampling size was repeated three times.

### Read Mapping and Assembling of the Chinese Amphioxus Genome

We used the RePS pipeline to improve the assembly result of the Chinese amphioxus. We used blastall 2.2.18 (-e 0.00001 -F F -v 1000 -b 1000) to map the BAC ends to the genome, and used a PERL script to obtain the top-hit result. Then, results whose alignment identity was less than 90% or alignment length less than 35 nt were discarded. For those reads that mapped to more than one place, we chose the best one as uniquely mapped. Then, we ran the RePS pipeline with default settings. The read pairs that mapped to different scaffolds were used to assemble the genome; the minimum number of scaffold links we used was two to make a valid connection. To obtain the span distribution, we also mapped the non-redundant read pairs to the genome, and read pairs that mapped to the same scaffold were used to estimate the average span.

## Supporting Information

Figure S1 **Information about paired BAC ends mapped to the Chinese amphioxus (** ***Branchiostoma belcheri*** **).**
(PDF)

Figure S2 **Length distribution of paired-end reads.**
(PDF)
