# Supplementary material for: HTS-PEG: A Method for High Throughput Sequencing of the Paired-Ends of Genomic Libraries
Source: PLoS One. 2012 Dec 20;7(12):e52257. doi: 10.1371/journal.pone.0052257 (PMC3527410; doi:10.1371/journal.pone.0052257)
Supplement: Figure S1 — Information about paired BAC ends mapped to the Chinese amphioxus ( Branchiostoma belcheri ). (PDF) [file pone.0052257.s001.pdf]

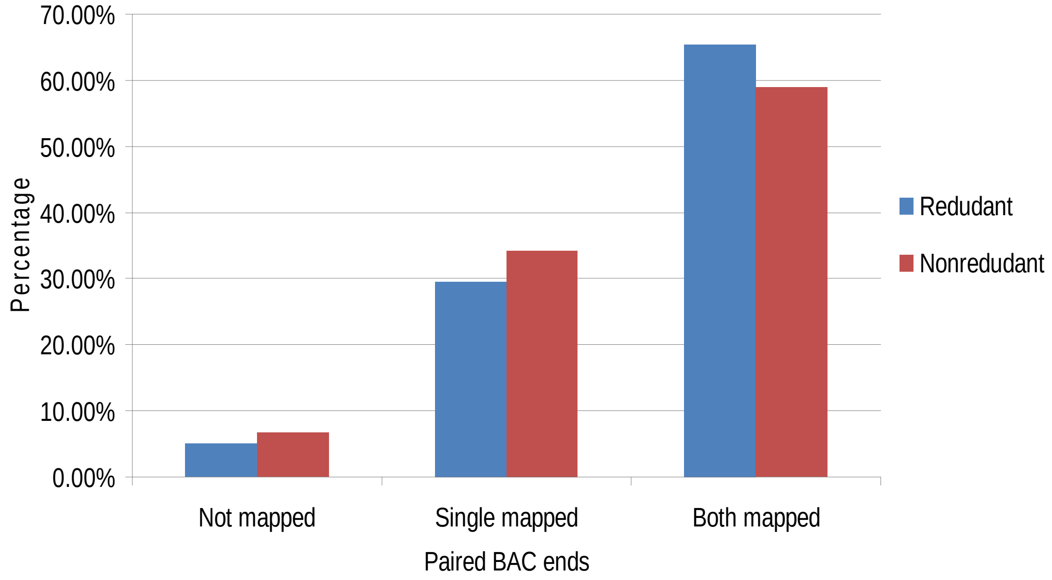

Supplementary Figure 1. Information about paired BAC ends mapped to the Chinese amphioxus (*Branchiostoma belcheri*).
