# Supplementary figures and images for: HTS-PEG: A Method for High Throughput Sequencing of the Paired-Ends of Genomic Libraries
Source: PLoS One. 2012 Dec 20;7(12):e52257. doi: 10.1371/journal.pone.0052257 (PMC3527410; doi:10.1371/journal.pone.0052257)

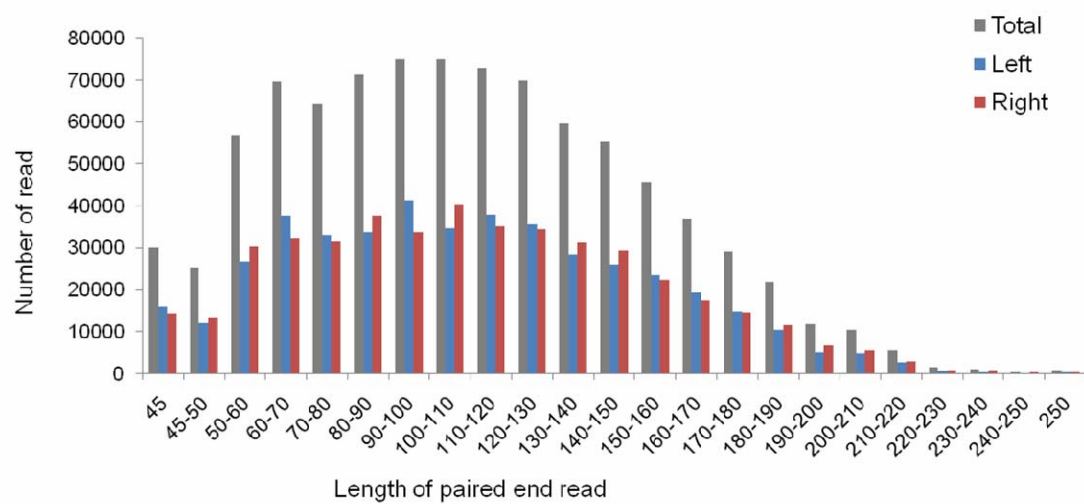

**Supplementary Figure 2.** Length distribution of paired-end reads.

Supplement: Figure S2 — Length distribution of paired-end reads. (PDF) [file pone.0052257.s002.pdf]
